# Effective Use of Virtual Gamification During COVID-19 to Deliver the OB-GYN Core Curriculum in an Emergency Medicine Resident Conference

**DOI:** 10.7759/cureus.8397

**Published:** 2020-06-01

**Authors:** Alanna O'Connell, Peter J Tomaselli, Megan Stobart-Gallagher

**Affiliations:** 1 Emergency Medicine, Thomas Jefferson University, Philadelphia, USA; 2 Emergency Medicine, Thomas Jefferson University Hospital, Philadelphia, USA

**Keywords:** medical education, gamification, emergency medicine resident, ob-gyn, core curriculum

## Abstract

Introduction

Coronavirus disease 2019 (COVID-19) has challenged medical educators on continuing to provide quality educational content in a virtual setting. The objective of this module was to create a gamified review of core obstetric and gynecology (OB-GYN) topics that residents would find educational and informative.

Methods

The game created was modeled after the TV show “So You Think You Can Dance?”, with a warm-up and several rounds of rapid-fire OB-GYN questions and cases, eliminating teams to a final face-off. The residents were given a post-session survey to determine their attitudes and learning towards this virtual conference approach.

Results

Based on the post-session survey, the majority of the residents found this activity to be educational, entertaining, engaging, and better than the traditional lecture format.

Conclusion

This initial attempt at migrating gamification, a core component of our live conferences, into the new virtual arena, was well-received by learners as effective, educational, and engaging. This style of gamification can be incorporated into residency programs at other institutions currently limited to virtual platforms to boost resident education and engagement.

## Introduction

One of the many challenges that arose during the coronavirus disease 2019 (COVID-19) healthcare crisis was maintaining the traditional format of non-clinical emergency medicine (EM) resident physician education. Given the social distancing regulations in place, traditional, weekly, in-person didactic sessions became difficult, if not impossible. Many residency programs across the country turned to virtual platforms to host weekly conferences and grand rounds [1]. At our institution, we moved the emergency medicine resident conference to an online video conferencing platform. While we were able to translate lectures and discussions relatively easily to this platform, we were missing one of the mainstays of our institutional education methods: active learning and gamification.

Gamification is an increasingly popular method of educational instruction that incorporates game mechanics into educational programs. It has been found to be more effective for improving knowledge, skills, and satisfaction as compared to traditional education methods in many studies [2]. There are many applications and virtual games available for medical education but, to our knowledge, there have been no published reports of virtual gamification attempted during a video conferencing version of weekly resident conferences [3]. With this in mind, we developed a novel virtual game for our obstetrics and gynecology (OB-GYN) module.

## Materials and methods

This educational innovation using gamification was designed for all post-graduate year (PGY) levels in emergency medicine (EM) at Thomas Jefferson University Hospital, a three-year EM training program located in Philadelphia, PA. Thirty-six PGY1-PGY3 residents participated in the exercise with one primary facilitator and six additional faculty members. It was executed using a synchronous, virtual format based upon the Zoom conferencing platform, which allows for breakout rooms. Breakout rooms can be randomly or manually assigned by the host of the conference and allow for small groups to that "break out" from the whole. 

**Figure 1:**
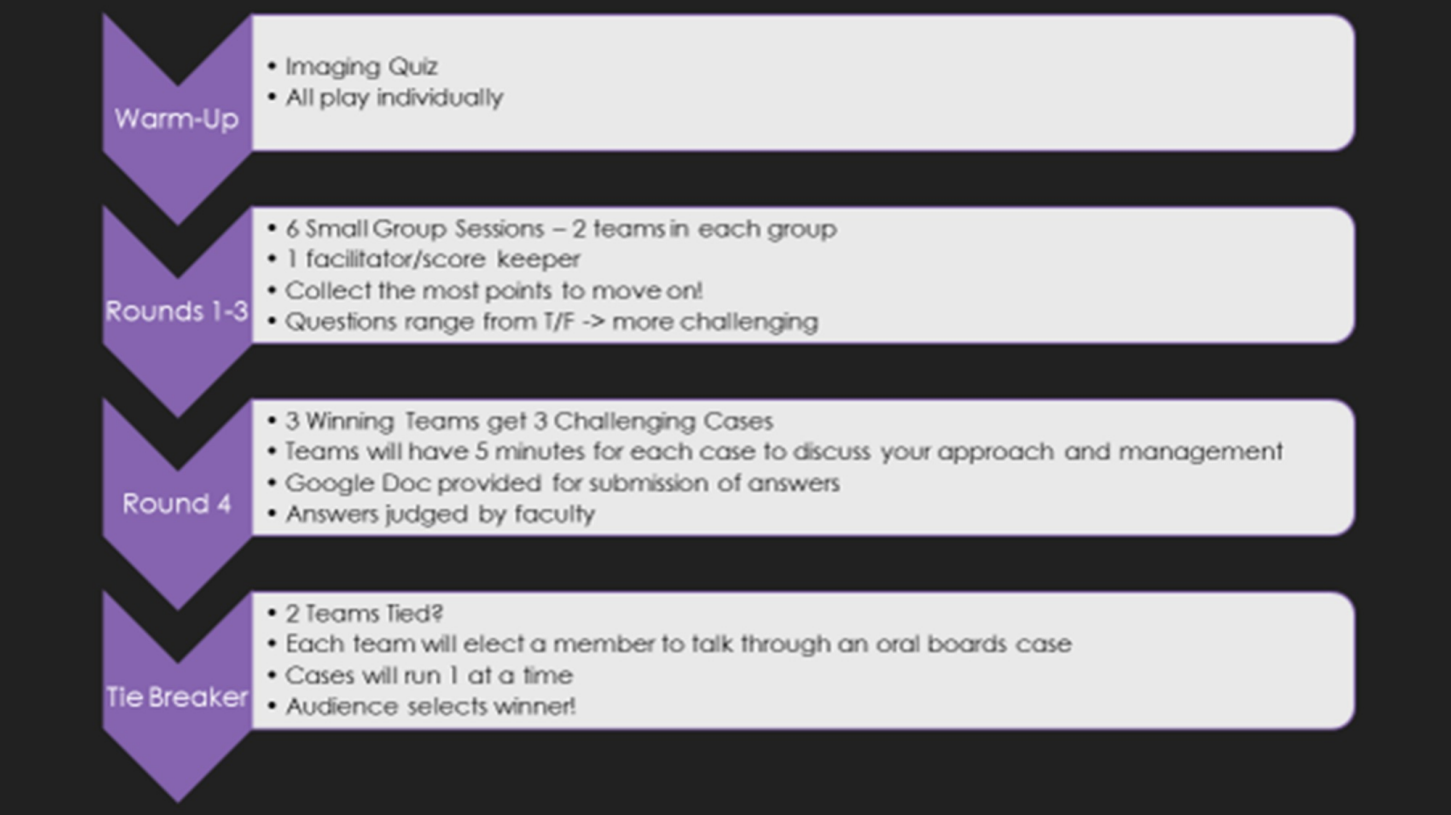
Format of the game

The game was formatted to emulate the reality TV show: "So You Think You Can Dance," with multiple rounds eliminating teams to a final face-off. In the theme of dancing and to entice their competitive spirit, all residents participated individually in the ‘warm-up’ round, using a Kahoot^TM^ (Oslo, Norway) quiz to test their knowledge of ultrasound (US) imaging in females. Each round focused on testing the team’s knowledge of a different aspect of obstetric and gynecological care. The detailed game structure is highlighted in Figure 1. The residents were divided into small groups and placed into a breakout room with a faculty facilitator who then divided their residents into two teams of three to four residents. The two teams then competed in this breakout room for the first three rounds.

Content for the rounds was taken from multiple sources, including core emergency medicine textbooks like Tintinalli’s Emergency Medicine and Rosen’s Emergency Medicine: Concepts and Clinical Practice and free open access medicine resources found to contain high-yield, vetted EM content via Google search [4-7]. The questions in Round 1 focused on the nonpregnant patient and questions were based on causes of dysfunctional bleeding and sexually transmitted and urinary tract infection management. Round 2 focused on the pregnant patient (i.e. the physiologic changes of pregnancy, pharmacologic doses and safety in pregnancy, and a “grab bag” of other obstetrical topics). Round 3 focused on labor and delivery and included topics such as risk factors for placental abruption, management of prolapsed cord, APGAR (Appearance, Pulse, Grimace, Activity, and Respiration) scoring for newborns, and neonatal resuscitation. After the first three rounds, scores were tabulated and the winning team from each group advanced to the fourth round, leaving a total of six teams to fight for the win.

For the fourth round, the breakout rooms were closed and everyone was brought back to the main conference room. This fourth and final round was a series of three cases in which the remaining teams were given a case and they had to write down their proposal for how each case should be managed into a Google Form, which was then submitted for judging. The three cases they were given to manage were postpartum hemorrhage, shoulder dystocia, and breech delivery. They were sent to “team” breakout rooms while the remaining residents were kept in the large group with the facilitators to discuss and review what they had learned so far. The fourth round was judged by the faculty facilitators - who considered the accuracy and appropriateness of management plans in their scoring - ultimately voting for their top two teams, with one team winning in a landslide.

In the case of a tie between the top two teams, a tiebreaker round was also prepared. In this tiebreaker, each team would have to elect a team member to run a mock oral boards case in front of their peers, with the ultimate winner then voted on by their peers. 

The participants were asked to complete an anonymous post-session survey via an online survey to ascertain the efficacy of this activity for learning. The survey used a five-point Likert scale to gauge the learners’ enjoyment, engagement, learning, and if they felt this format was more effective than traditional lectures. There was also an opportunity for narrative comments and constructive feedback at the end. As this was an educational innovation and not a research study, this study was considered exempt by the Institutional Review Board.

## Results

**Figure 2:**
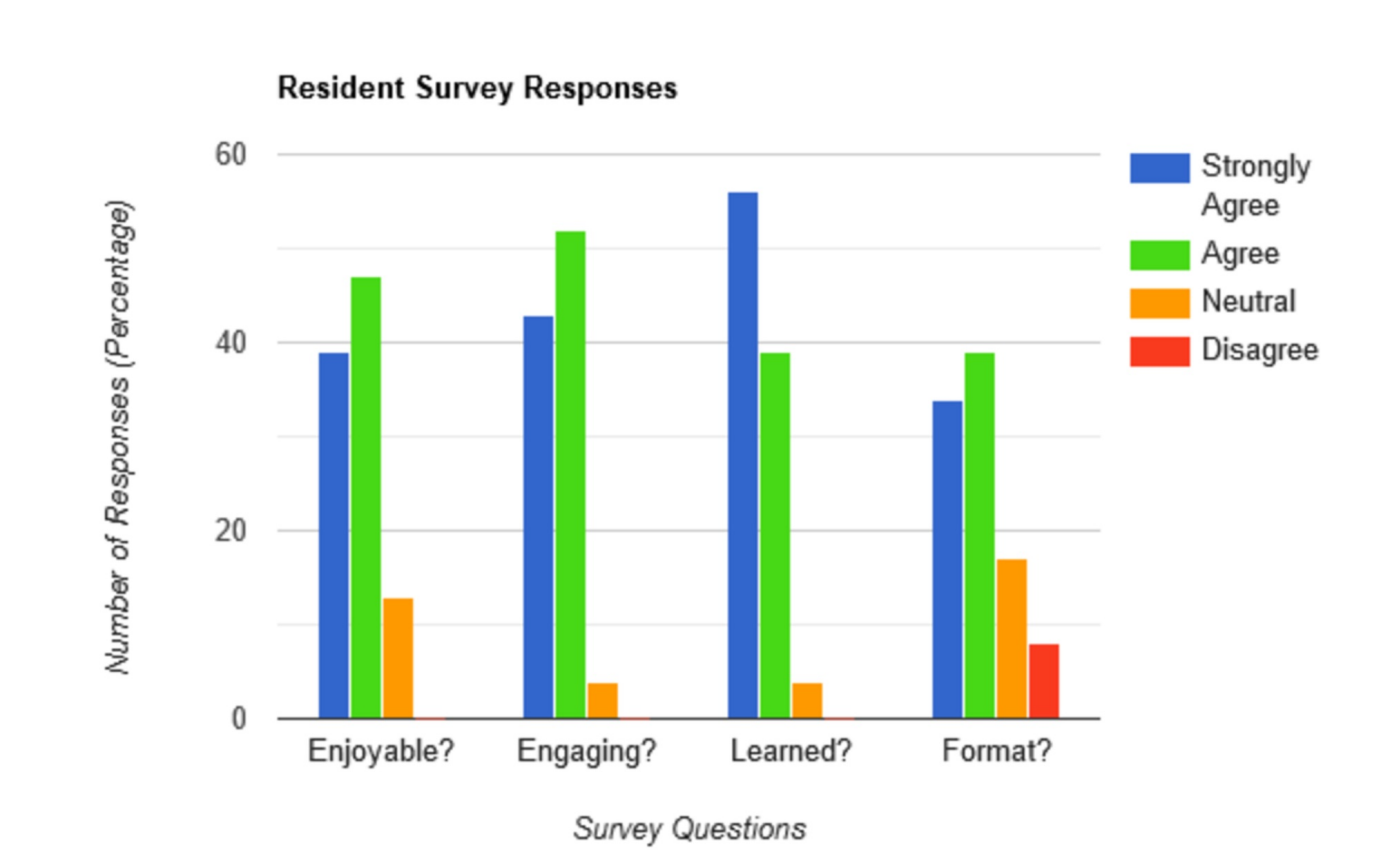
Resident survey responses

Out of the 36 residents in attendance, 23 completed an anonymous online survey for a return rate of 63%. Their responses, as shown in Figure 2, demonstrated that a large majority of the residents enjoyed the activity, with only 13% giving a neutral response. Ninety-five percent of residents were in agreement or strong agreement that they were engaged during the activity. Five percent felt neutral to their engagement and no residents felt disengaged. All residents were either neutral or in agreement in regards to learning something new from this session. Lastly, residents were asked if they felt this activity was better than a traditional lecture for reviewing the material presented. Seventy-four percent were in agreement or strongly agreed that this was better, with 17% neutral and 9% in disagreement, preferring traditional lectures.

Free text responses for comments and constructive feedback were also accepted, and mostly included positives such as “fun and engaging” and “small group sessions were fun and interactive”; however, they also included one comment stating that “the games in the group didn’t work well, small group discussion would work better.”

## Discussion

Overall, the experience was well-received by the learners in attendance. The majority of responses to the surveyed areas were positive, with them finding the activity to be enjoyable, educational, engaging, and better received when compared to the traditional lecture format. The comments at the end of the survey reflected this as well.

The only question that received some constructive feedback was the question regarding if the game was better than lectures for reviewing the material, and 8.7% of residents disagreed that the game was an improvement over traditional lectures. One comment for feedback also suggested that this resident would prefer a different format for learning as well. Given what we know in educational theory regarding the learning styles of different residents, it would make sense that this format would not appeal to all residents [8].

The facilitators were also invited to provide lessons learned and constructive feedback for this gamification innovation. While each facilitator did receive instructions on how to run and score their group’s teams, interpretations of the instructions varied, leading to execution inconsistencies. Each group appeared to run slightly differently with the timing of questions, who in each group would answer questions, or if the teams would go head-to-head for each individual question. We also learned that this type of innovation requires more time than originally slated, leading some teams to not finish all of the questions/sections. Within the virtual format - we also found some challenges with communicating with the facilitators once they were in their breakout rooms. This may be a limitation of technology or the overall facilitation of the session. We would improve these perceived issues in the future by having a pregame meeting with all facilitators to improve training and solidify instructions, adding co-hosts to troubleshoot technological issues, and performing a run-through of the game before introducing learners.

There were some limitations to our study. This is a single-center study with data from a single classroom session resulting in a relatively low number of participants. We received post-session surveys from 23 of the 36 residents participating for a return rate of 64%. Our post-session survey also focused on resident’s attitudes towards the game and was not able to assess its educational efficacy in a concrete way (i.e. in-training examination scores, etc.). For future attempts at virtual gamification, one could look at pre/post tests containing educational content to assess if the format truly was effective for teaching the material. A larger sample size could be obtained following incorporation at other residency programs.

## Conclusions

The novel coronavirus pandemic has significantly changed the face of medicine during this time, and it has disrupted the usual didactic education of resident physicians. To maintain the quality of our educational systems as much as possible, it is important to try to maintain consistency with our department's core curriculum and ideals. This initial attempt at migrating gamification, a core component of our live conferences, into the new virtual arena was well-received by learners as effective, educational, and engaging. This style of gamification can be incorporated into residency programs at other institutions currently limited to virtual platforms to boost resident education and engagement.

## Appendices

Post-Session Questionnaire:

1: You enjoyed this conference activity, mark only one:

Strongly Disagree

Disagree

Neutral

Agree

Strongly agree

2. You felt engaged in learning during this session, mark only one:

Strongly Disagree

Disagree

Neutral

Agree

Strongly Agree

3. You learned something new from this session, mark only one:

Strongly Disagree

Disagree

Neutral

Agree

Strongly Agree

4. A relay race game was better than lecture for reviewing material, mark only one:

Strongly Disagree

Disagree

Neutral

Agree

Strongly Agree

5. Do you have any suggestions for improvement or comments in general on this session?
